# Backscatter-Aided Relaying for Interactive Dual-HAP Wireless-Powered Sensor Networks

**DOI:** 10.3390/s26123916

**Published:** 2026-06-20

**Authors:** Yuan Zheng, Haisong Chen, Huan Wan, Yongxue Wang

**Affiliations:** 1 School of Integrated Circuits, Shenzhen Polytechnic University, Shenzhen 518055, China; 2 Undergraduate School of Artificial Intelligence, Shenzhen Polytechnic University, Shenzhen 518055, China; 3 Shenzhen Key Laboratory of Media Security, Guangdong Provincial Key Laboratory of Intelligent Information Processing, Shenzhen University, Shenzhen 518060, China

**Keywords:** wireless-powered sensor networks, wireless-powered transfer, backscatter communication, cooperative relaying

## Abstract

This paper investigates backscatter-aided relaying for interactive dual-HAP wireless-powered sensor networks (WPSNs), in which two cooperative sensor groups transmit sensed data to opposite hybrid access points (HAPs) using harvested radio-frequency energy. Each group consists of multiple source sensor nodes (SNs) and one relay SN selected according to its proximity to the target HAP. To reduce local cooperation overhead, source SNs reuse the wireless power transfer (WPT) signal as a controllable carrier and convey their information to the relay SN through passive backscatter communication. The collected information is then delivered to the target HAPs through direct source transmission and relay forwarding. A source common-throughput maximization problem is formulated by jointly optimizing time allocation, transmit energy allocation, and dual-HAP energy beamforming, subject to energy-causality and relay minimum-rate constraints. To address the resulting non-convexity, an alternating optimization algorithm is developed, where the time-and-energy allocation subproblem is transformed into a convex form and the energy beamforming matrices are updated through energy-feasibility margin maximization. Numerical results show that the proposed scheme outperforms active cooperation without backscatter and direct transmission, demonstrating the effectiveness of integrating passive local information collection, relay-assisted uplink transmission, and optimized dual-HAP WPT.

## 1. Introduction

Wireless-powered sensor networks (WPSNs) have emerged as a promising solution for sustainable sensing and data collection in energy-constrained Internet-of-Things (IoT) applications [1,2,3,4]. In WPSNs, sensor nodes harvest radio-frequency energy from hybrid access points (HAPs) via downlink (DL) wireless power transfer (WPT), and then use the harvested energy for uplink (UL) wireless information transmission (WIT). This harvest-then-transmit operation reduces the need for battery replacement, but its performance is strongly affected by the coupled DL energy transfer and UL data transmission processes. Due to distance-dependent attenuation, far sensor nodes usually harvest less energy while requiring more transmit energy for data delivery, leading to the doubly near-far problem [5,6]. In multi-sensor scenarios, this energy–information imbalance may severely limit the source common throughput and fairness performance. Therefore, efficient transmission protocols and resource allocation strategies are needed for energy-constrained sensor nodes. In this context, learning-based approaches have also been explored for adaptive protocol design in large-scale wireless networks, including multi-agent reinforcement learning for generalized MAC protocol learning [7].

Relay-assisted cooperation has been widely used to improve throughput fairness in wireless-powered networks. A two-user cooperative WPCN was studied in [6], where one user forwards the information of the other to the AP. In [8], cooperating WDs were enabled to form a distributed virtual antenna array for joint UL transmission in a WPCN with separated energy and information nodes. Relay selection and transceiver design for cooperative SWIPT networks were investigated in [9]. More recently, In [10], the authors extended cooperation to a multi-user WPSN through cluster-based information collection and forwarding. These studies confirm the benefit of cooperative relaying, but they generally rely on active source-to-relay information exchange before forwarding. Such local cooperation consumes additional time and harvested energy, which is particularly restrictive in WPSNs with energy-limited source and relay nodes.

Backscatter communication provides an energy-efficient approach for local information exchange in WPSNs. By modulating and reflecting an incident RF signal, a sensor node can convey information without generating an active RF carrier. A comprehensive survey of ambient backscatter communication was presented in [11], and its application to RF-powered cognitive radio networks was studied in [12]. Signal detection and performance analysis for ambient backscatter systems were further investigated in [13,14,15]. These studies demonstrate the feasibility of low-power backscatter transmission. However, conventional ambient backscatter usually depends on opportunistic RF sources with uncertain availability and signal strength. In WPSNs, the DL WPT signal from HAPs can serve as a controllable carrier for source-to-relay information collection.

The controllable WPT signal also enables backscatter communication to be integrated with cooperative transmission. In [16], an energy-beacon-powered backscatter-aided relay system was studied. Backscatter-aided relaying was further investigated in [17], where the source backscatters information to the relay and receiver before active forwarding. In [18], backscatter communication was integrated with a harvest-then-transmit operation in a two-user WPCN. Furthermore, the authors of [19] investigated a multi-backscatter WPCN for green IoT and optimized its resource allocation to improve energy efficiency. To more clearly position the proposed scheme, Table 1 compares it with four representative backscatter-assisted wireless-powered designs in terms of network architecture, the role of backscatter communication, cooperative relaying, energy beamforming, and optimization objective. As summarized in Table 1, the representative schemes mainly consider multi-relay, two-user, single-HAP, or multi-backscatter settings. The joint coordination of DL WPT beamforming, backscatter-based multi-source information collection, active source transmission, and relay forwarding in interactive dual-HAP WPSNs has not been fully investigated.

**Figure 1:**
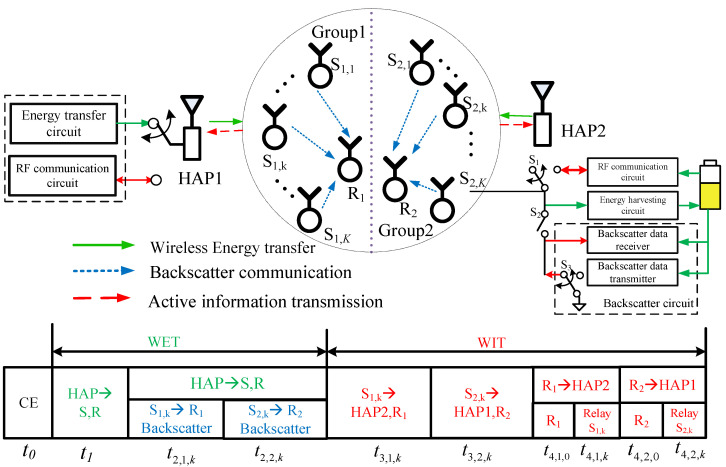
The schematic illustration of the system model and transmission protocol for the proposed cooperation scheme.

Motivated by the above discussions, this paper studies backscatter-aided relaying in an interactive dual-HAP WPSN. As shown in Figure 1, two cooperative sensor groups transmit their data to opposite HAPs. Each group consists of multiple source nodes and one relay node selected according to its proximity to the target HAP. The source nodes first reuse the incident DL WPT signal to convey their information to the relay through passive backscatter communication. The collected information is then delivered to the target HAPs through direct source transmission and relay forwarding. By embedding local information collection into the wireless-powered transmission process, this protocol reduces the time and energy overhead of conventional active cooperation. The source common throughput is maximized by jointly optimizing the time allocation, transmit energy allocation, and dual-HAP energy beamforming, subject to energy-causality constraints and relay minimum-rate requirements.

The main contributions are summarized as follows.

- A backscatter-aided relaying protocol is developed for interactive dual-HAP WPSNs. The DL WPT signal is reused as a carrier for passive source-to-relay information collection, avoiding active transmit energy consumption in the local cooperation phase. Based on this protocol, the harvested energy and achievable throughput are characterized, and a source common-throughput maximization problem is formulated.
- An AO-based algorithm is proposed to solve the resulting non-convex problem. For fixed energy beamforming matrices, the time and transmit energy allocation subproblem is reformulated into a convex problem through auxiliary transmit energy variables. For the obtained allocation, the energy beamforming matrices are updated by maximizing the energy-feasibility margin, which preserves feasibility and yields a non-decreasing objective value.
- Numerical results validate the advantage of the proposed design under different network settings. Compared with active cooperation without backscatter and direct transmission, the proposed scheme achieves higher source common throughput. The gain is mainly attributed to the joint use of passive local information collection, relay-assisted UL WIT, and optimized dual-HAP WPT.

The remainder of this paper is organized as follows. Section 2 presents the system model and transmission protocol. Section 3 analyzes the harvested energy and achievable throughput. Section 4 formulates the source common-throughput maximization problem and develops the AO-based solution. Section 5 provides numerical results and performance comparisons. Section 6 concludes this paper.

## 2. System Model

### 2.1. Channel Model

As shown in Figure 1, we consider an interactive dual-HAP WPSN consisting of two multi-antenna hybrid access points (HAPs) and multiple single-antenna sensor nodes (SNs). The two HAPs, denoted by HAP1 and HAP2, have stable energy supplies and are equipped with *M* antennas. Each SN relies on harvested RF energy to support active information transmission. Each SN is equipped with an energy harvesting circuit, a passive backscatter circuit, and an active RF communication circuit [11]. Accordingly, source SNs can harvest energy, backscatter information by reusing the incident WPT signal, and actively transmit data to the target HAP. The selected relay SN further decodes the backscattered source information and actively transmits both its own sensed data and the decoded source information.

The SNs are assigned to two cooperative groups, $G_{1}$ and $G_{2}$, corresponding to two predefined sensing regions in the considered interactive dual-HAP WPSN. The SNs in $G_{1}$ and $G_{2}$ transmit their sensed data to HAP2 and HAP1, respectively. Given this group association, the SN closest to the corresponding target HAP is selected as the relay SN in each group, while the remaining SNs act as source SNs. Each group contains *K* source SNs and one relay SN. The source SNs and relay SN in $G_{i}$ are denoted by $S_{i,k}$, k=1,…,K, and $R_{i}$, respectively. In each group, the SN closest to the target HAP is selected as the relay SN, and the remaining SNs act as source SNs. Thus, $R_{1}$ and $R_{2}$ are selected according to their proximity to HAP2 and HAP1, respectively. This rule is adopted because the relay SN is responsible for both forwarding the source information and transmitting its own sensed data to the target HAP.

For the HAP-to-SN links, let $a_{jS_{i,k}}\in C^{M\times 1}$ denote the channel vector from HAP *j* to source SN $S_{i,k}$, where j=1,2, i=1,2, and k=1,…,K. Similarly, $a_{jR_{i}}\in C^{M\times 1}$ denotes the channel vector from HAP *j* to relay SN $R_{i}$. Channel reciprocity is assumed under TDD operation, so the corresponding UL channels are represented by the same channel vectors.

The channel power gains of the HAP-to-source-SN and HAP-to-relay-SN links are respectively defined as

(1) $$h_{jS_{i,k}}={∥a_{jS_{i,k}}∥}^{2},h_{jR_{i}}={∥a_{jR_{i}}∥}^{2},j=1,2,i=1,2,k=1,\ldots ,K.$$

In particular, $h_{2S_{1,k}}$ and $h_{2R_{1}}$ characterize the links from $G_{1}$ to its target HAP, HAP2, while $h_{1S_{2,k}}$ and $h_{1R_{2}}$ characterize the links from $G_{2}$ to HAP1.

For the intra-group source-to-relay links, let $c_{i,k}$ denote the channel coefficient between source SN $S_{i,k}$ and relay SN $R_{i}$. The corresponding channel power gain is

(2) $$g_{i,k}={\left|c_{i,k}\right|}^{2},i=1,2,k=1,\ldots ,K.$$

These intra-group links are used for backscatter-aided source-to-relay information collection. Channel reciprocity is also assumed for the intra-group links.

All channels are mutually independent and follow quasi-static flat fading. The channel coefficients remain unchanged within one transmission block of duration *T*, and vary independently across different blocks.

### 2.2. Protocol Description

We consider a block-based transmission protocol with duration *T*, as shown in Figure 1. A fixed duration $t_{0}$ is reserved for channel estimation (CE). The remaining block is used for DL WPT, backscatter-based source-to-relay information collection, active source transmission, and relay transmission. During the DL WPT interval $t_{1}$, HAP1 and HAP2 simultaneously broadcast RF energy signals to all sensor nodes with energy beamforming. The harvested energy is stored for subsequent active transmission, while the WPT signals are also reused as incident carriers for backscatter communication. Then, the source nodes transmit their information to the corresponding relay nodes through passive backscatter in a TDMA manner. Specifically, $S_{1,k}$ backscatters to $R_{1}$ during $t_{2,1,k}$, and $S_{2,k}$ backscatters to $R_{2}$ during $t_{2,2,k}$, where k=1,…,K. In this process, the source nodes do not generate dedicated RF carriers, and the relay nodes decode the backscattered information while continuing to harvest RF energy.

After the backscatter-based information collection, the source nodes actively transmit to their target HAPs using the harvested energy. Source node $S_{1,k}$ transmits to HAP2 during $t_{3,1,k}$, while $S_{2,k}$ transmits to HAP1 during $t_{3,2,k}$. The target HAPs employ maximum-ratio combining (MRC) for UL signal reception. The relay nodes may overhear this interval, but only the Phase-II backscatter signals are used for relay-side source information decoding. Finally, the relay nodes transmit to their target HAPs. Relay node $R_{1}$ transmits to HAP2, and $R_{2}$ transmits to HAP1. Since each relay node also has its own sensed data, $t_{4,1,0}$ and $t_{4,2,0}$ are allocated for the own-information transmission of $R_{1}$ and $R_{2}$, respectively. The forwarding times for the source information are denoted by $t_{4,1,k}$ and $t_{4,2,k}$.

Accordingly, the overall time allocation satisfies

(3) $$t_{0}+t_{1}+\sum_{k=1}^{K}\left(t_{2,1,k}+t_{3,1,k}+t_{4,1,k}\right)+\sum_{k=1}^{K}\left(t_{2,2,k}+t_{3,2,k}+t_{4,2,k}\right)+t_{4,1,0}+t_{4,2,0}\leq T.$$

The block duration is normalized as T=1 in the following analysis for simplicity.

## 3. Throughput Performance Analysis

In this section, we characterize the harvested energy and achievable throughput of the proposed backscatter-aided relaying protocol according to the four transmission phases in Section 2.2.

### 3.1. Phase I: Wireless Power Transfer

During Phase I, HAP1 and HAP2 simultaneously transmit RF energy signals to all SNs for duration $t_{1}$. Let $x_{j}\left(t\right)\in C^{M\times 1}$ denote the energy signal sent by HAP*j*, where j=1,2. The corresponding energy beamforming covariance matrix is defined as

(4) $$Q_{j}=E\left[x_{j}\left(t\right)x_{j}^{H}\left(t\right)\right],$$

with

(5) $$Q_{j}⪰0,\;\mathrm{tr}\left(Q_{j}\right)\leq P_{j},\;j=1,2.$$

For source SN $S_{i,k}$, the received RF signal is

(6) $$y_{S_{i,k}}^{\left(1\right)}\left(t\right)=a_{1S_{i,k}}^{H}x_{1}\left(t\right)+a_{2S_{i,k}}^{H}x_{2}\left(t\right)+n_{S_{i,k}}^{\left(1\right)}\left(t\right),i=1,2,k=1,\ldots ,K,$$

where $n_{S_{i,k}}^{\left(1\right)}\left(t\right)$ is the receiver noise. Similarly, the received signal at relay SN $R_{i}$ is

(7) $$y_{R_{i}}^{\left(1\right)}\left(t\right)=a_{1R_{i}}^{H}x_{1}\left(t\right)+a_{2R_{i}}^{H}x_{2}\left(t\right)+n_{R_{i}}^{\left(1\right)}\left(t\right).$$

The harvested energy from receiver noise is negligible, and the energy signals transmitted by the two HAPs are assumed to be independent. Define

(8) $$A_{jS_{i,k}}=a_{jS_{i,k}}a_{jS_{i,k}}^{H},\;\;A_{jR_{i}}=a_{jR_{i}}a_{jR_{i}}^{H},j=1,2,i=1,2,k=1,\ldots ,K.$$

Then, the incident RF power at source SN $S_{i,k}$ is

(9) $$A_{S_{i,k}}=\mathrm{tr}\left(A_{1S_{i,k}}Q_{1}\right)+\mathrm{tr}\left(A_{2S_{i,k}}Q_{2}\right),$$

and the harvested energy in Phase I is

(10) $$E_{S_{i,k}}^{\left(1\right)}=\eta t_{1}A_{S_{i,k}},$$

where η denotes the energy harvesting efficiency.

Likewise, the incident RF power and harvested energy at relay SN $R_{i}$ are respectively given by

(11) $$A_{R_{i}}=\mathrm{tr}\left(A_{1R_{i}}Q_{1}\right)+\mathrm{tr}\left(A_{2R_{i}}Q_{2}\right),$$

and

(12) $$E_{R_{i}}^{\left(1\right)}=\eta t_{1}A_{R_{i}}.$$

### 3.2. Phase II: Backscatter Communication

During Phase II, the source SNs deliver their information to the corresponding relay SNs through passive backscatter communication. The source SNs in each group are scheduled in a TDMA manner. Specifically, in group $G_{i}$, source SN $S_{i,k}$ backscatters its information to relay SN $R_{i}$ during $t_{2,i,k}$,i=1,2 and k=1,…,K.

The RF energy signals transmitted by HAP1 and HAP2 are reused as incident carriers for backscatter communication. Hence, the two HAPs continue transmitting energy signals with the same covariance matrices $Q_{1}$ and $Q_{2}$ as in Phase I. During the backscatter interval of $S_{i,k}$, the incident RF signal at $S_{i,k}$ is given by

(13) $$y_{S_{i,k}}^{\left(2\right)}\left(t\right)=a_{1S_{i,k}}^{H}x_{1}\left(t\right)+a_{2S_{i,k}}^{H}x_{2}\left(t\right)+n_{S_{i,k}}^{\left(2\right)}\left(t\right),$$

where $n_{S_{i,k}}^{\left(2\right)}\left(t\right)$ denotes the receiver noise at $S_{i,k}$.

Let $s_{b,i,k}\left(t\right)$ denote the unit-power backscatter information symbol of $S_{i,k}$, i.e., $E[|s_{b,i,k}\left(t\right){|}^{2}]=1$. Let $\mu_{i,k}$ be the power reflection coefficient with $0\leq \mu_{i,k}\leq 1$. Then, the backscattered signal generated by $S_{i,k}$ can be modeled as

(14) $$b_{i,k}^{\left(2\right)}\left(t\right)=\sqrt{\mu_{i,k}}\;s_{b,i,k}\left(t\right)y_{S_{i,k}}^{\left(2\right)}\left(t\right).$$

Substituting $y_{S_{i,k}}^{\left(2\right)}\left(t\right)$ into the above expression yields

(15) $$b_{i,k}^{\left(2\right)}\left(t\right)=\sqrt{\mu_{i,k}}\;s_{b,i,k}\left(t\right)\left(a_{1S_{i,k}}^{H}x_{1}\left(t\right)+a_{2S_{i,k}}^{H}x_{2}\left(t\right)+n_{S_{i,k}}^{\left(2\right)}\left(t\right)\right).$$

The received signal at relay SN $R_{i}$ during the backscatter interval of $S_{i,k}$ is

(16) $$\begin{array}{cc} y_{R_{i},k}^{\left(2\right)}\left(t\right)= & c_{i,k}b_{i,k}^{\left(2\right)}\left(t\right)+a_{1R_{i}}^{H}x_{1}\left(t\right)+a_{2R_{i}}^{H}x_{2}\left(t\right)+n_{R_{i}}^{\left(2\right)}\left(t\right), \end{array}$$

where $c_{i,k}$ is the channel coefficient from $S_{i,k}$ to $R_{i}$, and $n_{R_{i}}^{\left(2\right)}\left(t\right)$ is the receiver noise at $R_{i}$. Equivalently, we have

(17) $$\begin{array}{c} y_{R_{i},k}^{\left(2\right)}\left(t\right)=\sqrt{\mu_{i,k}}c_{i,k}s_{b,i,k}\left(t\right)\left(a_{1S_{i,k}}^{H}x_{1}\left(t\right)+a_{2S_{i,k}}^{H}x_{2}\left(t\right)\right)+a_{1R_{i}}^{H}x_{1}\left(t\right)+a_{2R_{i}}^{H}x_{2}\left(t\right)\\ +\sqrt{\mu_{i,k}}c_{i,k}s_{b,i,k}\left(t\right)n_{S_{i,k}}^{\left(2\right)}\left(t\right)+n_{R_{i}}^{\left(2\right)}\left(t\right). \end{array}$$

In the above expression, the first term carries the backscatter information, whereas the next two terms are the direct RF energy signals from the HAPs. The reflected noise is much weaker than the receiver noise after passive reflection and inter-SN propagation, and is neglected in both information decoding and energy harvesting.

Since the same energy beamforming matrices are used in Phases I and II, the incident RF powers at $S_{i,k}$ and $R_{i}$ remain $A_{S_{i,k}}$ and $A_{R_{i}}$, respectively. With $g_{i,k}={\left|c_{i,k}\right|}^{2}$, the reflected signal power received at $R_{i}$ is modeled as $\mu_{i,k}g_{i,k}A_{S_{i,k}}$. Thus, the energy harvested by relay SN $R_{i}$ during the backscatter interval of $S_{i,k}$ is

(18) $$E_{R_{i,k}}^{\left(2\right)}=\eta t_{2,i,k}\left(A_{R_{i}}+\mu_{i,k}g_{i,k}A_{S_{i,k}}\right).$$

By summing over all source SNs in $G_{i}$, the total energy harvested by relay SN $R_{i}$ in Phase II is

(19) $$E_{R_{i}}^{\left(2\right)}=\eta \sum_{k=1}^{K}t_{2,i,k}\left(A_{R_{i}}+\mu_{i,k}g_{i,k}A_{S_{i,k}}\right).$$

Following the effective backscatter transmission model, the amount of information collected by relay SN $R_{i}$ from source SN $S_{i,k}$ is expressed as

(20) $$R_{S_{i,k}\to R_{i}}^{\left(2\right)}=C_{i,k}R_{b}t_{2,i,k},$$

where $R_{b}$ is the hardware-dependent nominal backscatter transmission rate, and $C_{i,k}$ denotes an effective capacity coefficient that captures the combined effects of backscatter propagation, synchronization, detection, and decoding conditions [16,17,18].

Therefore, the total energy available at relay SN $R_{i}$ after Phases I and II is

(21) $$E_{R_{i}}=E_{R_{i}}^{\left(1\right)}+E_{R_{i}}^{\left(2\right)},\;i=1,2.$$

This energy is used by $R_{i}$ for its own information transmission and source-information forwarding in Phase IV.

### 3.3. Phase III: Active Source Transmission

After backscatter-based information collection, the source SNs actively transmit their information to the target HAPs. Specifically, $S_{1,k}$ transmits to HAP2 during $t_{3,1,k}$, while $S_{2,k}$ transmits to HAP1 during $t_{3,2,k}$, where k=1,…,K. Since the HAPs are equipped with multiple antennas, maximum-ratio combining (MRC) is adopted for UL reception [20]. Let $P_{3,1,k}$ and $P_{3,2,k}$ denote the transmit powers of $S_{1,k}$ and $S_{2,k}$, respectively. The corresponding transmit energy constraints are

(22) $$\begin{array}{c} t_{3,1,k}P_{3,1,k}\leq E_{S_{1,k}}^{\left(1\right)}=\eta t_{1}A_{S_{1,k}},\;t_{3,2,k}P_{3,2,k}\leq E_{S_{2,k}}^{\left(1\right)}=\eta t_{1}A_{S_{2,k}}. \end{array}$$

Let $x_{3,1,k}\left(t\right)$ and $x_{3,2,k}\left(t\right)$ denote the unit-power information-bearing signals transmitted by $S_{1,k}$ and $S_{2,k}$, respectively. When $S_{1,k}$ transmits to HAP2, the received signal vector is

(23) $$y_{2,k}^{\left(3\right)}\left(t\right)=a_{2S_{1,k}}\sqrt{P_{3,1,k}}x_{3,1,k}\left(t\right)+n_{2,k}^{\left(3\right)}\left(t\right),$$

where $n_{2,k}^{\left(3\right)}\left(t\right)∼\mathrm{CN}\left(0,N_{0}I\right)$ is the AWGN vector at HAP2. With MRC, the received SNR is

(24) $$\gamma_{S_{1,k}\to \mathrm{HAP}2}^{\left(3\right)}=\frac{h_{2S_{1,k}}P_{3,1,k}}{N_{0}}.$$

Thus, the achievable rate is

(25) $$R_{S_{1,k}\to \mathrm{HAP}2}^{\left(3\right)}=t_{3,1,k}\log_{2}\left(1+\frac{h_{2S_{1,k}}P_{3,1,k}}{N_{0}}\right).$$

Similarly, when $S_{2,k}$ transmits to HAP1, the received signal vector is

(26) $$y_{1,k}^{\left(3\right)}\left(t\right)=a_{1S_{2,k}}\sqrt{P_{3,2,k}}x_{3,2,k}\left(t\right)+n_{1,k}^{\left(3\right)}\left(t\right),$$

where $n_{1,k}^{\left(3\right)}\left(t\right)∼\mathrm{CN}\left(0,N_{0}I\right)$ is the AWGN vector at HAP1. With MRC, the received SNR is

(27) $$\gamma_{S_{2,k}\to \mathrm{HAP}1}^{\left(3\right)}=\frac{h_{1S_{2,k}}P_{3,2,k}}{N_{0}}.$$

The achievable rate is therefore

(28) $$R_{S_{2,k}\to \mathrm{HAP}1}^{\left(3\right)}=t_{3,2,k}\log_{2}\left(1+\frac{h_{1S_{2,k}}P_{3,2,k}}{N_{0}}\right).$$

Although the relay SNs may overhear the active source signals due to wireless broadcast propagation, these side observations are not exploited in the proposed protocol. The relay-side source information is obtained only from Phase-II backscatter communication. Hence, Phase III contributes only to the direct source-to-target-HAP information recovery.

### 3.4. Phase IV: Relay Transmission

In Phase IV, relay SNs transmit both their own sensed data and the decoded source information to the target HAPs. Specifically, $R_{1}$ transmits to HAP2 and $R_{2}$ transmits to HAP1. Thus, the relay transmission phase consists of own-information transmission and source-information forwarding. For $R_{1}$, let $t_{4,1,0}$ and $P_{4,1,0}$ denote the time and transmit power allocated to its own-information transmission. The received signal vector at HAP2 is

(29) $$y_{2,0}^{\left(4\right)}\left(t\right)=a_{2R_{1}}\sqrt{P_{4,1,0}}x_{4,1,0}\left(t\right)+n_{2,0}^{\left(4\right)}\left(t\right),$$

where $x_{4,1,0}\left(t\right)$ is the unit-power information-bearing signal of $R_{1}$, and $n_{2,0}^{\left(4\right)}\left(t\right)∼\mathrm{CN}\left(0,N_{0}I\right)$ is the AWGN vector at HAP2. With MRC reception, the achievable rate is

(30) $$R_{R_{1}\to \mathrm{HAP}2}^{\left(4\right)}=t_{4,1,0}\log_{2}\left(1+\frac{h_{2R_{1}}P_{4,1,0}}{N_{0}}\right).$$

Then, $R_{1}$ forwards the decoded information of source SN $S_{1,k}$ to HAP2 during $t_{4,1,k}$ with transmit power $P_{4,1,k}$. The received signal vector is

(31) $$y_{2,k}^{\left(4\right)}\left(t\right)=a_{2R_{1}}\sqrt{P_{4,1,k}}x_{4,1,k}\left(t\right)+n_{2,k}^{\left(4\right)}\left(t\right),$$

where $x_{4,1,k}\left(t\right)$ is the unit-power re-encoded signal carrying the information of $S_{1,k}$. The corresponding forwarding rate is

(32) $$R_{S_{1,k}\to \mathrm{HAP}2}^{\left(4\right)}=t_{4,1,k}\log_{2}\left(1+\frac{h_{2R_{1}}P_{4,1,k}}{N_{0}}\right).$$

Similarly, relay SN $R_{2}$ transmits its own information to HAP1 during $t_{4,2,0}$ with transmit power $P_{4,2,0}$, yielding

(33) $$R_{R_{2}\to \mathrm{HAP}1}^{\left(4\right)}=t_{4,2,0}\log_{2}\left(1+\frac{h_{1R_{2}}P_{4,2,0}}{N_{0}}\right).$$

When $R_{2}$ forwards the decoded information of $S_{2,k}$ to HAP1 during $t_{4,2,k}$ with transmit power $P_{4,2,k}$, the forwarding rate is

(34) $$R_{S_{2,k}\to \mathrm{HAP}1}^{\left(4\right)}=t_{4,2,k}\log_{2}\left(1+\frac{h_{1R_{2}}P_{4,2,k}}{N_{0}}\right).$$

The relay transmission energy cannot exceed the energy harvested by the relay SNs in Phases I and II. Hence, the energy-causality constraints of $R_{1}$ and $R_{2}$ are given by

(35) $$\begin{array}{c} t_{4,1,0}P_{4,1,0}+\sum_{k=1}^{K}t_{4,1,k}P_{4,1,k}\leq E_{R_{1}},\;t_{4,2,0}P_{4,2,0}+\sum_{k=1}^{K}t_{4,2,k}P_{4,2,k}\leq E_{R_{2}}. \end{array}$$

For each source SN, the end-to-end throughput is limited by the information collected at the relay in Phase II and the information recovered at the target HAP through direct source transmission and relay forwarding. Thus, the achievable throughput of $S_{1,k}$ is

(36) $$R_{S_{1,k}}=\min\left\{R_{S_{1,k}\to R_{1}}^{\left(2\right)},R_{S_{1,k}\to \mathrm{HAP}2}^{\left(3\right)}+R_{S_{1,k}\to \mathrm{HAP}2}^{\left(4\right)}\right\},$$

and that of $S_{2,k}$ is

(37) $$R_{S_{2,k}}=\min\left\{R_{S_{2,k}\to R_{2}}^{\left(2\right)},R_{S_{2,k}\to \mathrm{HAP}1}^{\left(3\right)}+R_{S_{2,k}\to \mathrm{HAP}1}^{\left(4\right)}\right\}.$$

Since the relay SNs also transmit their own sensed data, their self-information rates are required to satisfy

(38) $$R_{R_{1}\to \mathrm{HAP}2}^{\left(4\right)}\geq R_{\min},\;R_{R_{2}\to \mathrm{HAP}1}^{\left(4\right)}\geq R_{\min}.$$

## 4. Max–Min Throughput Optimization

### 4.1. Problem Formulation

In this section, we jointly optimize the time allocation, active transmit-power allocation, and dual-HAP energy beamforming matrices for the proposed backscatter-aided relaying scheme. The objective is to maximize the source common throughput among all source SNs. Since the relay SNs also transmit their own sensed data, minimum-rate constraints are imposed to guarantee their individual transmission requirements.

The optimization problem is formulated as

(39) $$\begin{array}{cc} \left(P1\right):\;\max_{t,P,Q_{1},Q_{2}}\;\; & \min_{k=1,\ldots ,K}\left(R_{S_{1,k}},R_{S_{2,k}}\right)\\ s.t.\;\; & (3),(10),(21),(22),\mathrm{and}\;(35),\\ \; & Q_{j}⪰0,\;\mathrm{tr}\left(Q_{j}\right)\leq P_{j},\;j=1,2,\\ \; & t_{1},t_{2,i,k},t_{3,i,k},t_{4,i,k},t_{4,i,0}\geq 0,\\ \; & P_{3,i,k},P_{4,i,k},P_{4,i,0}\geq 0,\;i=1,2,\;k=1,\ldots ,K,\\ \; & R_{R_{1}\to \mathrm{HAP}2}^{\left(4\right)}\geq R_{\min},\\ \; & R_{R_{2}\to \mathrm{HAP}1}^{\left(4\right)}\geq R_{\min}. \end{array}$$

where $t=[t_{1},t_{2,i,k},t_{3,i,k},t_{4,i,0},t_{4,i,k}]$ collects all time allocation variables, and $P=[P_{3,i,k},P_{4,i,0},P_{4,i,k}]$ collects the active transmit-power variables of the source and relay SNs. The matrices $Q_{1}$ and $Q_{2}$ denote the energy beamforming covariance matrices at HAP1 and HAP2, respectively. The source throughput $R_{S_{i,k}}$ is determined by the end-to-end expression involving Phase-II backscatter collection, Phase-III direct source transmission, and Phase-IV relay forwarding.

The objective in (P1) represents the source common throughput and characterizes the fairness among all source SNs in the two cooperative groups. The relay minimum-rate constraints reflect the dual role of the relay SNs, which not only forward source information but also transmit their own sensed data. Problem (P1) is non-convex due to the coupled time–power products, the max–min objective, and the interaction between energy beamforming and harvested-energy constraints. An AO-based solution is developed in the next subsection.

### 4.2. Alternating Optimization Solution

Problem (P1) is non-convex because the time allocation, transmit-power allocation, and energy beamforming matrices are tightly coupled in both the rate expressions and the energy-causality constraints. To solve this problem, an alternating optimization (AO) algorithm is developed. For fixed energy beamforming matrices, the time and transmit energy allocation are optimized through a convex reformulation. For the obtained allocation, the energy beamforming matrices are then updated by maximizing the energy-feasibility margin.

#### 4.2.1. Optimizing Time and Transmit Energy Allocation

For given $Q_{1}$ and $Q_{2}$, the incident RF powers $A_{S_{i,k}}$ and $A_{R_{i}}$ are fixed. To handle the products between time and transmit power, define the transmit energy variables as

(40) $$\tau_{3,i,k}=t_{3,i,k}P_{3,i,k},\;\tau_{4,i,0}=t_{4,i,0}P_{4,i,0},\;\tau_{4,i,k}=t_{4,i,k}P_{4,i,k},$$

where i=1,2 and k=1,…,K.

With these variables, the Phase-III source transmission rates become

(41) $$R_{S_{1,k}\to \mathrm{HAP}2}^{\left(3\right)}=t_{3,1,k}\log_{2}\left(1+\frac{h_{2S_{1,k}}\tau_{3,1,k}}{t_{3,1,k}N_{0}}\right),$$

(42) $$R_{S_{2,k}\to \mathrm{HAP}1}^{\left(3\right)}=t_{3,2,k}\log_{2}\left(1+\frac{h_{1S_{2,k}}\tau_{3,2,k}}{t_{3,2,k}N_{0}}\right).$$

Similarly, the Phase-IV relay rates are rewritten as

(43) $$R_{R_{1}\to \mathrm{HAP}2}^{\left(4\right)}=t_{4,1,0}\log_{2}\left(1+\frac{h_{2R_{1}}\tau_{4,1,0}}{t_{4,1,0}N_{0}}\right),$$

(44) $$R_{S_{1,k}\to \mathrm{HAP}2}^{\left(4\right)}=t_{4,1,k}\log_{2}\left(1+\frac{h_{2R_{1}}\tau_{4,1,k}}{t_{4,1,k}N_{0}}\right),$$

(45) $$R_{R_{2}\to \mathrm{HAP}1}^{\left(4\right)}=t_{4,2,0}\log_{2}\left(1+\frac{h_{1R_{2}}\tau_{4,2,0}}{t_{4,2,0}N_{0}}\right),$$

(46) $$R_{S_{2,k}\to \mathrm{HAP}1}^{\left(4\right)}=t_{4,2,k}\log_{2}\left(1+\frac{h_{1R_{2}}\tau_{4,2,k}}{t_{4,2,k}N_{0}}\right).$$

The source energy-causality constraints are expressed as

(47) $$\tau_{3,i,k}\leq \eta t_{1}A_{S_{i,k}},\;i=1,2,\;k=1,\ldots ,K.$$

For given $Q_{1}$ and $Q_{2}$, the harvested energy at relay SN $R_{i}$ is an affine function of $t_{1}$ and $t_{2,i,k}$, i.e.,

(48) $$E_{R_{i}}=\eta t_{1}A_{R_{i}}+\eta \sum_{k=1}^{K}t_{2,i,k}\left(A_{R_{i}}+\mu_{i,k}g_{i,k}A_{S_{i,k}}\right),\;i=1,2.$$

Thus, the relay energy-causality constraints become

(49) $$\tau_{4,1,0}+\sum_{k=1}^{K}\tau_{4,1,k}\leq E_{R_{1}},\tau_{4,2,0}+\sum_{k=1}^{K}\tau_{4,2,k}\leq E_{R_{2}}.$$

By introducing an auxiliary variable $\bar{R}$, the time-and-transmit energy allocation subproblem for fixed $Q_{1}$ and $Q_{2}$ is formulated as

$$\begin{array}{cc} \left(P2\right):\;\max_{\bar{R},t,\tau }\;\; & \bar{R}\\ s.t.\;\; & t_{0}+t_{1}+\sum_{k=1}^{K}\left(t_{2,1,k}+t_{3,1,k}+t_{4,1,k}\right)\\ \; & \;+\sum_{k=1}^{K}\left(t_{2,2,k}+t_{3,2,k}+t_{4,2,k}\right)+t_{4,1,0}+t_{4,2,0}\leq 1,\\ \; & t_{1},t_{2,i,k},t_{3,i,k},t_{4,i,k},t_{4,i,0}\geq 0,i=1,2,\\ \; & \tau_{3,i,k},\tau_{4,i,k},\tau_{4,i,0}\geq 0,i=1,2,\\ \; & \tau_{3,1,k}\leq \eta t_{1}A_{S_{1,k}},\tau_{3,2,k}\leq \eta t_{1}A_{S_{2,k}},\;k=1,\ldots ,K,\\ \; & \tau_{4,1,0}+\sum_{k=1}^{K}\tau_{4,1,k}\leq E_{R_{1}},\\ \; & \tau_{4,2,0}+\sum_{k=1}^{K}\tau_{4,2,k}\leq E_{R_{2}},\\ \; & \bar{R}\leq C_{1,k}R_{b}t_{2,1,k},\;k=1,\ldots ,K,\\ \; & \bar{R}\leq C_{2,k}R_{b}t_{2,2,k},\;k=1,\ldots ,K,\\ \; & \bar{R}\leq R_{S_{1,k}\to \mathrm{HAP}2}^{\left(3\right)}+R_{S_{1,k}\to \mathrm{HAP}2}^{\left(4\right)},\;k=1,\ldots ,K,\\ \; & \bar{R}\leq R_{S_{2,k}\to \mathrm{HAP}1}^{\left(3\right)}+R_{S_{2,k}\to \mathrm{HAP}1}^{\left(4\right)},\;k=1,\ldots ,K,\\ \; & R_{R_{1}\to \mathrm{HAP}2}^{\left(4\right)}\geq R_{\min},\\ \; & R_{R_{2}\to \mathrm{HAP}1}^{\left(4\right)}\geq R_{\min}. \end{array}$$

For fixed $Q_{1}$ and $Q_{2}$, all energy-causality constraints in (P2) are affine in t and τ. The rate terms have the form $x\log_{2}\left(1+ay/x\right)$, which is the perspective of a logarithmic function and is jointly concave in (x,y) for x≥0 and y≥0. Therefore, the constraints involving $\bar{R}$ are convex superlevel constraints of concave rate functions, while the remaining constraints are linear. Hence, (P2) is a convex optimization problem and can be efficiently solved by standard convex optimization tools.

#### 4.2.2. Optimizing Energy Beamforming

After solving (P2), the time allocation t and transmit energy allocation τ are fixed. Under fixed t and τ, the rate expressions are determined, while the energy beamforming matrices still affect the feasibility of the source and relay energy-causality constraints. Therefore, $Q_{1}$ and $Q_{2}$ are updated by maximizing the minimum energy-feasibility margin.

For any sensor node *X*, define the incident RF power under given energy beamforming matrices as

(50) $$A_{X}\left(Q_{1},Q_{2}\right)=\mathrm{tr}\left(A_{1X}Q_{1}\right)+\mathrm{tr}\left(A_{2X}Q_{2}\right).$$

Then, the total harvested energy at relay SN $R_{i}$ can be expressed as

(51) $$E_{R_{i}}\left(Q_{1},Q_{2}\right)=\eta t_{1}A_{R_{i}}\left(Q_{1},Q_{2}\right)+\eta \sum_{k=1}^{K}t_{2,i,k}\left[A_{R_{i}}\left(Q_{1},Q_{2}\right)+\mu_{i,k}g_{i,k}A_{S_{i,k}}\left(Q_{1},Q_{2}\right)\right],$$

where i=1,2. For given t and τ, the energy beamforming subproblem is formulated as

(52) $$\begin{array}{cc} \left(P3\right):\;\max_{\sigma ,Q_{1},Q_{2}}\;\; & \sigma \\ s.t.\;\; & Q_{j}⪰0,\;\mathrm{tr}\left(Q_{j}\right)\leq P_{j},\;j=1,2,\sigma \geq 0,\\ \; & \tau_{3,1,k}+\sigma \leq \eta t_{1}A_{S_{1,k}}\left(Q_{1},Q_{2}\right),\;k=1,\ldots ,K,\\ \; & \tau_{3,2,k}+\sigma \leq \eta t_{1}A_{S_{2,k}}\left(Q_{1},Q_{2}\right),\;k=1,\ldots ,K,\\ \; & \tau_{4,1,0}+\sum_{k=1}^{K}\tau_{4,1,k}+\sigma \leq E_{R_{1}}\left(Q_{1},Q_{2}\right),\\ \; & \tau_{4,2,0}+\sum_{k=1}^{K}\tau_{4,2,k}+\sigma \leq E_{R_{2}}\left(Q_{1},Q_{2}\right). \end{array}$$

where σ denotes the minimum energy margin among all source and relay energy-causality constraints. Since $A_{X}\left(Q_{1},Q_{2}\right)$ and $E_{R_{i}}\left(Q_{1},Q_{2}\right)$ are affine functions of $Q_{1}$ and $Q_{2}$, (P3) is a convex semidefinite-constrained optimization problem.

Based on the above two subproblems, the proposed AO algorithm is developed in Algorithm 1. Starting from feasible initial energy beamforming matrices, (P2) and (P3) are solved alternately until the improvement of the source common throughput is below ϵ. The convergence follows from the feasibility-preserving property of (P3). After (P2), the obtained time and transmit energy allocations are feasible under the current beamforming matrices. Since the current beamforming matrices with σ=0 are feasible for (P3), the updated beamforming matrices preserve this allocation. Therefore, the next solution of (P2) cannot decrease the objective value. With finite block duration and HAP transmit powers, the AO algorithm converges.

In each AO iteration, problem (P2) contains 10K+6 scalar variables and can be solved with an approximate complexity of $O(K^{3})$ using an interior-point method. Problem (P3) contains two M×M semidefinite matrix variables, and its complexity is approximately $O(M^{4.5})$. Therefore, the per-iteration complexity of the proposed AO algorithm is $O!\left(K^{3}+M^{4.5}\right).$ The optimization is performed at the HAP side and is suitable for centralized block-level resource allocation in small- and medium-scale WPSNs.
**Algorithm 1** AO-based algorithm for solving (P1)**Input:** Convergence threshold ϵ and initial energy beamforming matrices $Q_{1}^{\left(0\right)}$ and $Q_{2}^{\left(0\right)}$.**Output:** $Q_{1}^{*}$, $Q_{2}^{*}$, $t^{*}$, and $P^{*}$.1:Set ℓ=0 and initialize ${\bar{R}}^{\left(0\right)}=0$.2:**repeat**3:    Solve (P2) for given $Q_{1}^{\left(\ell \right)}$ and $Q_{2}^{\left(\ell \right)}$, and obtain $t^{\left(\ell +1\right)}$, $\tau^{\left(\ell +1\right)}$, and ${\bar{R}}^{\left(\ell +1\right)}$.4:    Solve (P3) for given $t^{\left(\ell +1\right)}$ and $\tau^{\left(\ell +1\right)}$, and obtain $Q_{1}^{\left(\ell +1\right)}$ and $Q_{2}^{\left(\ell +1\right)}$.5:    Set ℓ=ℓ+1.6:**until** ℓ≥1 and $|{\bar{R}}^{\left(\ell \right)}-{\bar{R}}^{\left(\ell -1\right)}|\leq \epsilon$7:Recover the source transmit powers as$$P_{3,i,k}^{*}=\frac{\tau_{3,i,k}^{*}}{t_{3,i,k}^{*}},\;i=1,2,\;k=1,\ldots ,K.$$8:Recover the relay transmit powers as$$P_{4,i,0}^{*}=\frac{\tau_{4,i,0}^{*}}{t_{4,i,0}^{*}},\;P_{4,i,k}^{*}=\frac{\tau_{4,i,k}^{*}}{t_{4,i,k}^{*}},\;i=1,2,\;k=1,\ldots ,K.$$9:**return** $Q_{1}^{*}$, $Q_{2}^{*}$, $t^{*}$, and $P^{*}$.

## 5. Simulation Results

This section presents numerical results for the proposed backscatter-aided relaying scheme in an interactive dual-HAP WPSN. All simulations and numerical optimizations were performed using MATLAB R2024b (The MathWorks, Inc., Natick, MA, USA) with the CVX package v2.2. The average source common throughput is used as the performance metric. Unless otherwise specified, HAP1 and HAP2 are located at (−8,0) m and (8,0) m, respectively, while the centers of $G_{1}$ and $G_{2}$ are located at (−4,0) m and (4,0) m. In each group, K+1 SNs are uniformly deployed within a circular region of radius *r*. The SN closest to the target HAP is selected as the relay SN, and the remaining *K* SNs act as source SNs.

For the HAP-to-SN links, the channel vector is generated as $a_{jX}∼\mathrm{CN}\left(0,\sigma_{jX}^{2}I\right)$, where the average channel power gain follows

(53) $$\sigma_{jX}^{2}=G_{A}{\left(\frac{3\times 10^{8}}{4\pi d_{jX}f_{c}}\right)}^{\lambda },$$

where $d_{jX}$ is the distance between HAP *j* and SN *X*, $f_{c}$ is the carrier frequency, $G_{A}$ is the antenna power gain, and λ is the path-loss exponent. The intra-group source-to-relay links are generated using the same distance-dependent path-loss model. Unless otherwise specified, the main simulation parameters are listed in Table 2. For each parameter setting, the results are averaged over 20 independent Monte Carlo trials, and 30 random SN deployments are generated in each trial to reduce the randomness of node locations and small-scale fading. The convergence threshold of the AO algorithm is set to $10^{-4}$ to ensure sufficient numerical accuracy without excessive iterations.

Two benchmark schemes are considered for comparison.

(1) Benchmark 1: Active cooperation without backscatter. Inspired by the active cooperation protocol in [10], this scheme adopts the same dual-HAP cooperative architecture as the proposed scheme, but replaces passive backscatter collection with orthogonal active source-to-relay transmission. The locally transmitted information is used only for relay decoding and is not combined at the target HAP. Hence, source SNs consume harvested energy for local information delivery. The time allocation, transmit energy allocation, and energy beamforming matrices are optimized under the corresponding energy-causality and relay minimum-rate constraints.

(2) Benchmark 2: Direct transmission. Following the conventional harvest-then-transmit protocol in WPCNs [5], this scheme removes relay-assisted cooperation. To keep the same group size, all K+1 SNs in each group directly transmit to their target HAPs after harvesting energy from the two HAPs. Thus, no source-to-relay information collection, relay forwarding, or relay minimum-rate constraint is involved. The energy beamforming matrices and time allocation are optimized according to the direct transmission protocol.

The two benchmarks are evaluated under the same dual-HAP topology, channel realizations, power budgets, SN deployments, and resource constraints as the proposed scheme. Benchmark 1 isolates the gain of backscatter-assisted local information collection by replacing it with active cooperation, while Benchmark 2 isolates the gain of relay forwarding by removing cooperative transmission. Hence, these protocol-matched benchmarks provide controlled comparisons of the two key mechanisms introduced in this work. For fairness, all schemes optimize their available resource variables according to their own transmission protocols. Thus, the comparison reflects the effects of backscatter-aided local information collection and relay-assisted UL transmission under the same network setting.

**Figure 2:**
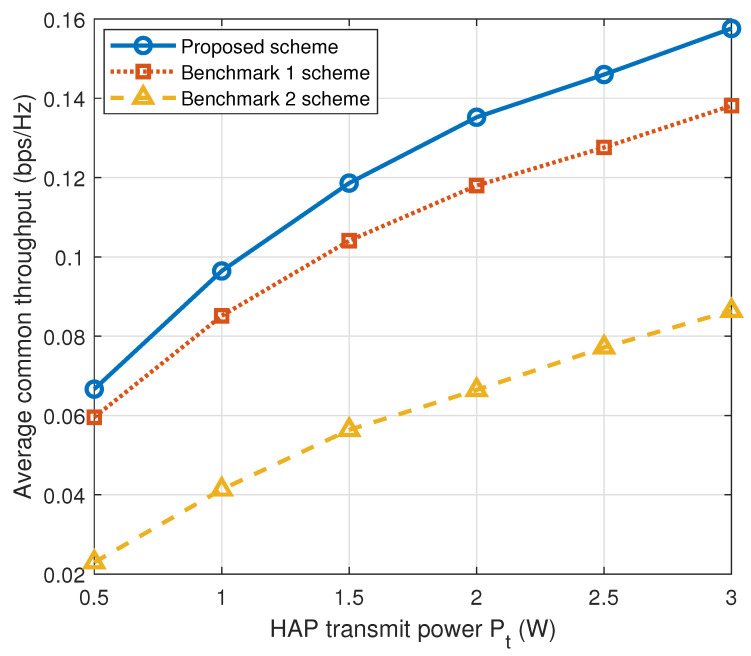
Average common throughput versus the HAP transmit power $P_{t}$.

Figure 2 plots the average source common throughput versus the HAP transmit power $P_{t}$, where $P_{1}=P_{2}=P_{t}$. A larger $P_{t}$ strengthens DL WPT and relaxes the energy-causality constraints, allowing more transmit energy for source transmission and relay forwarding. The proposed scheme achieves the highest throughput because passive source-to-relay information collection avoids active energy consumption at the source SNs. By contrast, active cooperation without backscatter spends part of the harvested energy on local collection, while direct transmission cannot exploit relay forwarding. Hence, the proposed scheme converts the increased WPT energy into cooperative UL throughput more efficiently.

**Figure 3:**
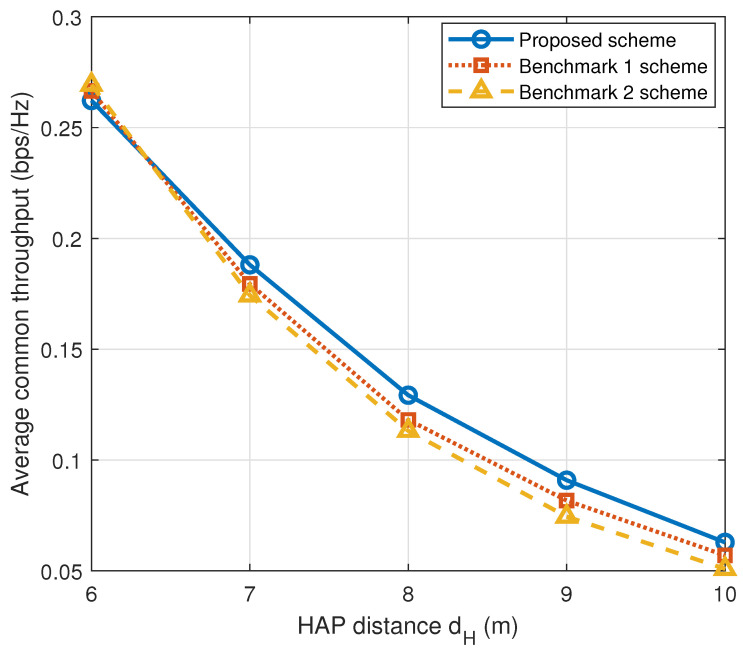
Average common throughput versus the HAP distance $d_{H}$.

Figure 3 shows the impact of the HAP distance parameter $d_{H}$. As $d_{H}$ increases, the source-to-HAP and relay-to-HAP WIT links become weaker, resulting in lower throughput for all schemes. The proposed scheme is more robust because passive backscatter preserves more harvested energy for effective UL transmission. Active cooperation without backscatter suffers from extra source energy consumption in local collection, whereas direct transmission relies only on weakened source-to-HAP links. This explains the performance advantage of the proposed scheme under larger HAP distances.

**Figure 4:**
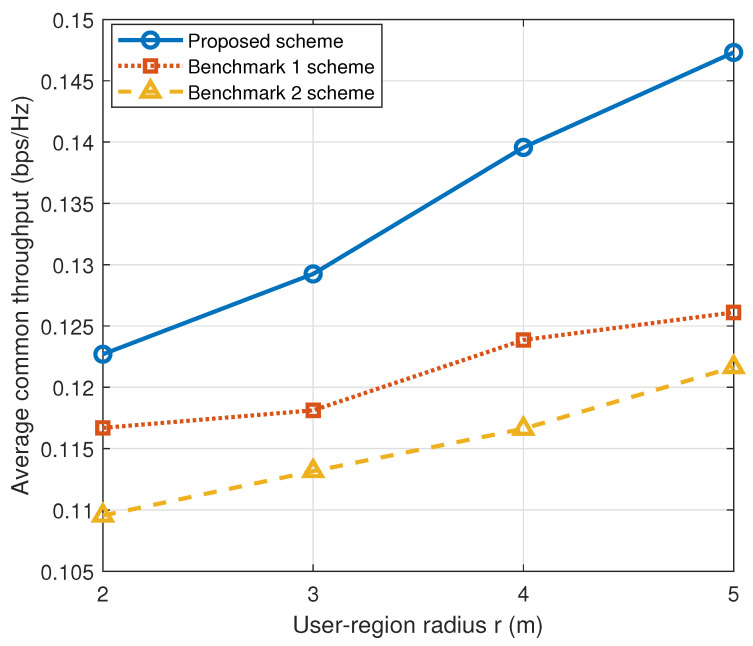
Average common throughput versus the user-region radius *r*.

Figure 4 examines the effect of the user-region radius *r*. A larger *r* increases the spatial diversity of SN locations and improves the probability of selecting a relay SN with a favorable relay-to-HAP link. Meanwhile, the energy beamforming matrices are re-optimized according to the updated node distribution, which enhances WET energy delivery to the selected source and relay SNs. The proposed scheme better converts this spatial flexibility into throughput gain because its source-to-relay information collection is performed through passive backscatter communication. Hence, source SNs avoid active transmit energy consumption in the local cooperation phase and reserve more harvested energy for source-to-HAP transmission and relay forwarding. By contrast, active cooperation without backscatter still consumes time and source energy for local collection, while direct transmission cannot exploit the selected relay for cooperative forwarding. On average, the proposed scheme improves the source common throughput by 11.01% and 16.75% compared with Benchmark 1 and Benchmark 2, respectively.

**Figure 5:**
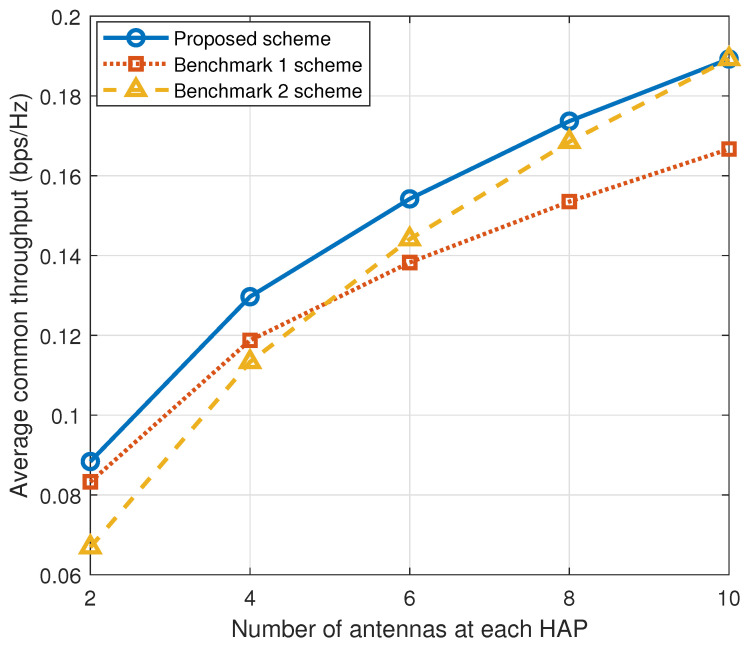
Average common throughput versus the number of HAP antennas.

Figure 5 shows the average source common throughput versus the number of HAP antennas *M*. Increasing *M* provides more spatial degrees of freedom for DL energy beamforming and improves the UL receive combining gain, thereby benefiting all schemes. The proposed scheme achieves the highest throughput because the multi-antenna WET and WIT gains are jointly exploited with passive local information collection and relay forwarding. The crossing between the two benchmarks is also reasonable. When *M* is small, the cooperation gain of active cooperation without backscatter is limited and may not compensate for its extra local collection overhead, making direct transmission competitive. As *M* increases, the improved relay-to-HAP link and relay energy harvesting enhance the value of cooperation, so Benchmark 1 gradually surpasses Benchmark 2.

**Figure 6:**
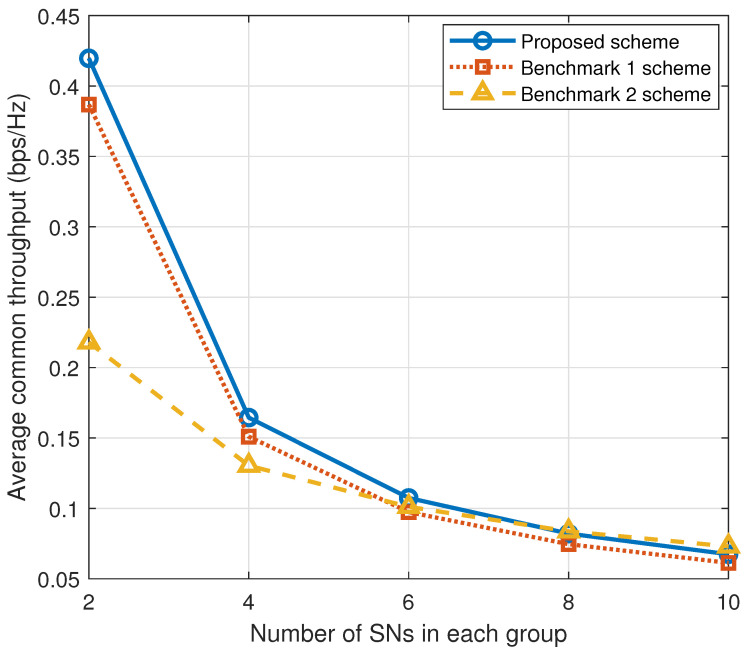
Average common throughput versus the number of SNs in each group.

Figure 6 evaluates the impact of the number of SNs in each group. As the group size increases, more source SNs share the fixed block duration and harvested energy, and the relay SN must support more forwarding tasks. Hence, the source common throughput decreases for all schemes. The proposed scheme maintains the best performance because passive backscatter reduces the time–energy cost of local source-to-relay information collection. In contrast, active cooperation without backscatter becomes more sensitive to the group size since each additional source SN introduces extra active local collection overhead. Direct transmission avoids this cooperation overhead and can therefore become competitive when the group size is large, but it lacks relay forwarding to compensate for weak source-to-HAP links.

**Figure 7:**
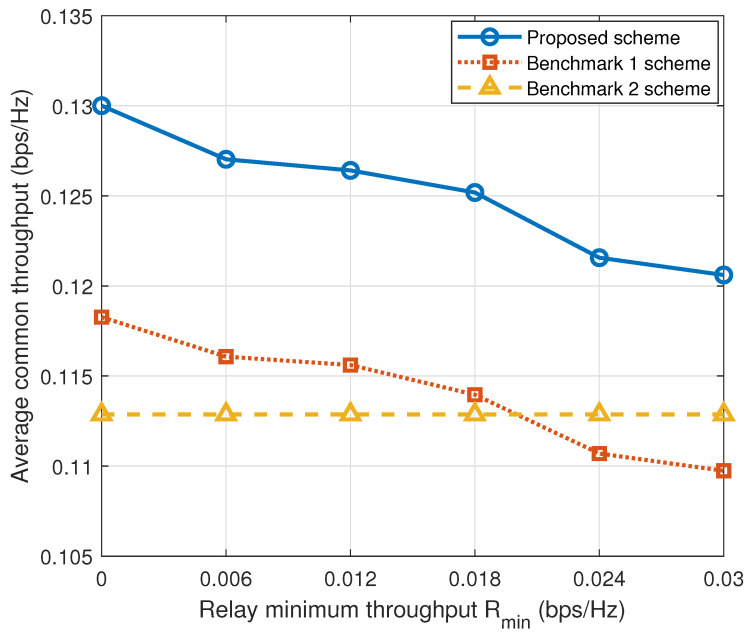
Average common throughput versus the relay minimum-rate requirement $R_{\min}$.

Figure 7 shows the effect of the relay minimum-rate requirement $R_{\min}$. For the two cooperative schemes, increasing $R_{\min}$ requires the relay SNs to allocate more time and transmit energy to their own data transmission, leaving fewer resources for source information forwarding. As a result, the source common throughput decreases. The proposed scheme is less affected because backscatter-based local collection preserves more harvested energy for relay transmission. Active cooperation without backscatter suffers from the additional source energy consumed before relay forwarding. By contrast, direct transmission has no relay role, and thus the relay minimum-rate constraint is not imposed on this scheme; its curve remains nearly unchanged and serves as a reference.

**Figure 8:**
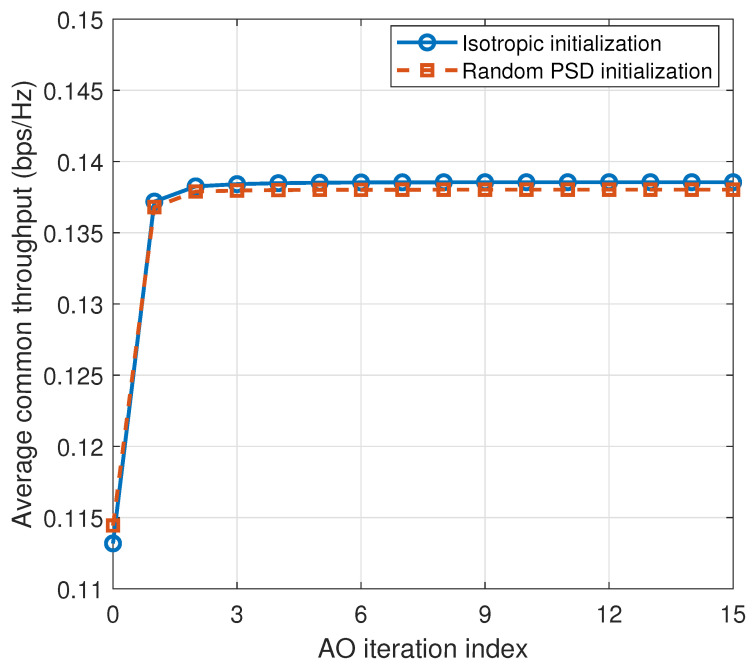
The convergence behavior of the proposed AO algorithm under different feasible initializations.

Figure 8 illustrates the average convergence behavior of the proposed AO algorithm under isotropic and random positive-semidefinite initializations. Under both initializations, the average common throughput increases rapidly during the first few iterations and becomes nearly unchanged afterward. The stopping criterion is satisfied within approximately four AO iterations, while the close final objective values indicate limited sensitivity to the tested initializations.

Overall, the results confirm that the proposed scheme achieves a better balance among DL WPT, local information collection, and cooperative UL WIT. By using passive backscatter for source-to-relay information collection, the source SNs avoid active transmit energy consumption in the local cooperation phase. Together with relay forwarding and optimized dual-HAP energy beamforming, the proposed scheme converts the available wireless energy and transmission time into a higher source common throughput than the benchmark schemes. The proposed protocol introduces additional requirements for dual-HAP coordination, CSI and scheduling-information exchange, block-level synchronization, and mode switching between active transmission and backscatter communication. The computational burden is mainly handled by the HAPs or a central controller rather than by the energy-constrained SNs. These additional implementation and coordination costs are incurred in exchange for avoiding active source-to-relay transmission and preserving more time and harvested energy for UL transmission. The required energy-harvesting, backscatter, and active RF modules are available in existing wireless-powered and backscatter hardware. However, a complete implementation still requires further system integration and control-signaling design.

## 6. Conclusions

This paper investigated backscatter-aided relaying for interactive dual-HAP WPSNs. In the proposed protocol, two cooperative sensor groups transmit data to opposite HAPs, where source SNs reuse the DL WPT signal for passive source-to-relay information collection, and the collected information is delivered through direct source transmission and relay forwarding. A source common-throughput maximization problem was formulated by jointly optimizing time allocation, transmit energy allocation, and dual-HAP energy beamforming under energy-causality and relay minimum-rate constraints. To solve the non-convex problem, an AO-based algorithm was developed by combining convex time-and-energy allocation with energy-feasibility-margin-based beamforming updates. Numerical results showed that the proposed scheme outperforms active cooperation without backscatter and direct transmission under different network settings. The gain mainly comes from reducing local cooperation overhead, preserving more harvested energy for relay-assisted UL transmission, and improving wireless energy delivery through optimized dual-HAP beamforming.

## Figures and Tables

**Table 1:** Comparison of the proposed scheme with representative backscatter-assisted wireless-powered communication schemes.

Work	Network Architecture	Role of Backscatter Communication	Cooperative Relaying	Energy Beamforming	Optimization Objective
Gong, S., et al. [16]	Multi-relay network with a multi-antenna HAP	Passive/active hybrid relaying	Yes	Yes	Throughput maximization
Lyu, B. et al. [17]	Single-HAP two-user WPCN	Passive information exchange for user cooperation	Yes	No	Throughput maximization
Zheng, Y. et al. [18]	Single-HAP backscatter-assisted WPCN	WPT-signal reuse for passive transmission	Yes	No	Common-throughput maximization
Gujral, S. et al. [19]	Multi-backscatter WPCN	Multi-device backscatter	Yes	Yes	Energy-efficiency maximization
Proposed scheme	Interactive dual-HAP multi-source WPSN	Passive multi-source information collection	Yes	Yes	Source common-throughput maximization

**Table 2:** Simulation parameters.

Parameter	Description	Value
$P_{t}$	HAP transmit power, $P_{1}=P_{2}=P_{t}$	2 W
*M*	Number of antennas at each HAP	4
*K*	Number of source SNs in each group	4
K+1	Total number of SNs in each group	5
η	Energy harvesting efficiency	0.6
$N_{0}$	Noise power	$10^{-10}$ W
$f_{c}$	Carrier frequency	915 MHz
λ	Path-loss exponent	2.5
$G_{A}$	Antenna power gain	3
$t_{0}$	Channel estimation time	0.05
$\mu_{i,k}$	Backscatter reflection coefficient	0.8
$C_{i,k}$	Effective backscatter coefficient	0.6
$R_{b}$	Nominal backscatter rate	10 bps/Hz
$R_{\min}$	Relay minimum-rate requirement	0.001 bps/Hz
*r*	User-region radius	3 m

## Data Availability

The data will be made available on request.
